# From Many Sources

**Published:** 1896-09

**Authors:** 


### FROM MANY SOURCES.

Polishing and coloring horns and hoofs of animals intended for show purposes is one of the latest fads.

A Wyoming County (N. Y.) cow has just been relieved of a butcher-knife which was extracted through the thoracic walls. The knife accidentally fell in the food a year ago, since which time it has been gradually working its way out.

J. L. Bradley, of Indianapolis, Ind., who owns the horse Thomas A. Scott, and has an extensive breeding farm near Edinburg, Ind., recently met with a severe loss. During an electric and rain storm lightning struck a bunch of brood-mares and youngsters at the farm, killing sixteen head.

In Colorado there are ruins five hundred years old on which there are rude sculptures of horses.

The muscles of the mocking-bird's larynx are larger in proportion to the size of the bird than those of any other creature.

In Australia horses and cattle are now being branded by electricity from storage batteries. The temperature is uniform and the brand safe and artistic.

Wild horses have increased to such an extent in Queensland that they are being shot with a view to reducing the number.

An Illinois Poland China boar pig has just changed hands at $1000.

The auditorium at St. Louis, where politics recently reigned supreme, will be converted into a sort of Madison Square Garden, for horse-shows, dog-shows, etc.

The New York City Board of Health, as a part of the municipal government, has recently had a verdict rendered against it for $1537.50 in favor of a livery-stable keeper, for five horses condemned and killed for glanders without being appraised by law as required.

